# Mackinawite formation from elemental iron and sulfur†

**DOI:** 10.1039/d1ra03705f

**Published:** 2021-10-01

**Authors:** Robert Bolney, Mario Grosch, Mario Winkler, Joris van Slageren, Wolfgang Weigand, Christian Robl

**Affiliations:** Faculty of Chemistry and Earth Sciences, Institute for Inorganic and Analytical Chemistry, Friedrich-Schiller-University Humboldtstrasse 8 07743 Jena Germany wolfgang.weigand@uni-jena.de christian.robl@uni-jena.de +49 3641 9-48160; Institute for Physical Chemistry, University of Stuttgart Pfaffenwaldring 55 70569 Stuttgart Germany

## Abstract

Sulfur-assisted corrosion is a process known to material scientists for many decades now. Though the corrosion of iron in the presence of sulfur has been studied extensively, it has never been used to intentionally synthesize mackinawite. In contrast to the conventional preparation of mackinawite by precipitation, the synthesis from the elements can be carried out without additional ions. This makes it possible to investigate the influence of any dissolved salts on the mackinawite formation and its properties. We found that the addition of NaCl significantly accelerates the reaction and furthermore influences the Fe^2+^ ion content of the formed mackinawite itself. This finding leads us to propose a novel model of charged layers which can be used to explain some of the inconsistencies found in the literature regarding the structure and particle characteristics of nano-mackinawite.

## Abstract 2

Nanoparticulate mackinawite was synthesized from elemental iron and sulfur in a convenient and reliable reaction. The structure and composition of the products were characterized and a new model describing the particle characteristics is established.

## Graphical abstract

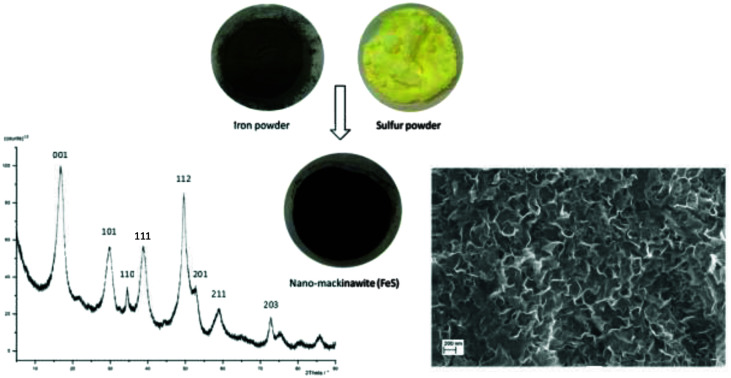

## Introduction

The fascinating, but almost forgotten reaction of forming iron sulfides from wet elemental iron and elemental sulfur under mild conditions was reported in 1700 AD, but this knowledge has not yet found its way into the inorganic chemistry textbooks.^1^ The reaction was again mentioned 200 years later in a short notice, but no characterization of the products was given.^2^ Inspired from these publications we have intensively investigated the reaction between elemental iron and elemental sulfur at ambient conditions to fill this gap.

During the last 60 years, the formation and characteristics of mackinawite have been studied from different points of view. Earth scientists have investigated mackinawite formation in laboratory and field studies to explore its role in natural biogeochemical processes. They mainly focused on the solubility and the transformation of precipitated mackinawite.^3–12^ Related to these studies are the investigations of mackinawite's pronounced capabilities to adsorb heavy and rare earth metalions what is of interest from many points of view.^13–15^ Organic chemists have studied its reducing power towards organic nitro and chlorinated compounds that may be useful for wastewater treatment.^16–18^ Its intrinsic physical properties like electrical conductivity and superconductivity have been studied by materials scientists. As a result, the intercalation chemistry of mackinawite has received some attention and experiments have been carried out to modify the interlayer space and to tune its electrical properties.^19–23^ Examinations of corrosion processes of iron in sulfide and sulfur containing solutions led to the discovery of mackinawite formation on metal surfaces. However, corrosion scientists have been more interested in the fate of the corroding metal than in the sulfides produced.^24–32^

The role of mackinawite has been discussed in the context of the origin of life and it could have contributed to crucial steps towards the emergence of the first self-replicating system. The oxidation of mackinawite coupled to the formation of pyrite has been studied extensively, due to its possible relevance as a primordial energy source as well as a very simple mimic of iron–sulfur proteins.^33–38^ Recently, Dzade *et al.* showed by DFT calculations that the mackinawite surface is able to activate CO_2_ molecules and to promote CO_2_ dissociation under certain conditions.^39,40^ The potential role of the pronounced adsorption ability of mackinawite was covered earlier by Picard and coworkers. Especially, amino acids, carbohydrates and lipids were captured by mackinawite particles and they concluded that “Biogenic iron sulfide minerals […] represent a potential strong protectant for proteinaceous organic carbon as long as anoxic conditions are preserved in low temperature surface environments”.^41^ The adsorption of biopolymer precursors increases their local concentrations and could have enabled polycondensation reactions. The capability of mackinawite to capture metal ions may have also led to a non-toxic environment in a stage, where biopolymers have formed which is one of “nine requirements” for the origin of earth's life as suggested by Maruyama *et al.* Toxic metal ions like Cu^2+^, Pb^2+^ and Hg^2+^ would have been precipitated in aqueous environments where mackinawite is present.^42^ Peng *et al.* reported on intercalation of cationic iron complexes of bipyridine, phenanthroline and other amino bases between mackinawite layers and such processes may also have contributed to condensation reactions on the prebiotic earth.^43^ Recently, the crystal structure of (C_2_H_8_N_2_)_0.4_Fe_2_S_2_ was published, which further supports this functionality.^44^ From these accounts it is apparent that the chemistry of iron sulfides with a mackinawite structure is a very interesting topic with much potential for various applications and doubtless many more features to discover.

The literature concerned with mackinawite, however, is full of inconsistencies regarding its composition, structure, stability, and reactivity (see Table 2). A useful reference for the structure and composition is the natural mineral as listed by the International Mineralogical Association (IMA). The composition was determined to be (Fe,Ni)_1+*x*_S (*x* = 0–0.07).^45^ This formula implies, that mackinawite is an iron sulfide with metal excess. Rickard *et al.* argue that the determination of the composition of mackinawite is quite difficult and that most analyses are erroneous. They analyzed a synthetic precipitated mackinawite sample and found a ratio of Fe : S very close to 1 : 1 without any water present in the particles. These results are in contrast to other reported analyses of synthetic mackinawite with compositions ranging from Fe_0.91_S to Fe_1.15_S.^46^ A detailed Rietveld investigation of the structure of synthetic mackinawite by Lennie *et al.* could also not reveal any excess iron or sulfur within the crystal structure.^47^ This Rietveld investigation is also commonly used as the reference for the structure of highly ordered mackinawite. They found that mackinawite has a layered structure with the space group *P*4/*nmm* and cell parameters of *a* = 3.6735(4) Å and *c* = 5.0328(7) Å. The value of the *c*-axis is the same as for the interlayer spacing and therefore corresponds to the *d*-value of the 001 diffraction peak of mackinawite.

In this work we investigate the unique formation and characteristics of mackinawite from elemental iron and sulfur at room temperature, following the publication from 1700 AD mentioned above.^1^ This method of synthesis offers the possibility to investigate the properties of mackinawite independently of the surrounding solution and at constant reagent concentrations.

## Experimental section

### Materials

The iron powder was bought from Sigma Aldrich (fine powder, reduced, ≥99%). It was manufactured by a reduction process using a reduction mixture consisting of coke breeze blended with ground limestone and a pre-processed magnetite slick. The sulfur powder and sodium chloride were bought from Sigma Aldrich and used as received. The deionized water used for the synthesis of mackinawite was purged with nitrogen for at least 12 hours and handled strictly under inert conditions.

### Methods

The mackinawite syntheses were carried out in 25 mL microwave vials with an aluminum cap and septum according to the following procedure: a finely ground mixture of powdered iron and powdered sulfur and any additional salts were placed in the vials and the vials were put under an inert nitrogen gas atmosphere. Using a syringe, 10 mL deoxygenated water was cautiously added without disturbing the iron–sulfur mixture. The vials were kept at room temperature or 80 °C for the assigned reaction times, and subsequently the products were isolated by filtration and dried in a nitrogen gas flow.

Powder X-ray diffraction analysis (PXRD) was carried out with a tabletop Rigaku Mini-Flex 600 equipped with a 0.6 kW copper anode X-ray source and an energy dispersive detector to minimize X-ray fluorescence effects.

A Rietveld refinement of powder diffraction data was carried out using the software “Topas 5” of Bruker^48^ using the mackinawite structure as published by Lennie *et al.*^49^ First, the instrument contribution to the PXRD pattern as well as the background function were determined with a LaB_6_ reference sample. Those were kept constant during further refinement. Any displacement along the *x*-axes was corrected based on the positions of residual sulfur and iron diffraction peaks.

Inductively coupled plasma atomic emission spectroscopy (ICP-AES) was carried out with a Varian 725 ES ICP optical emission spectrometer.

Scanning electron microscopy (SEM) imaging was performed with a Sigma VP Field Emission Scanning Electron Microscope (Carl-Zeiss AG, Germany) using an InLens detector with an accelerating voltage of 6 kV.

Energy-dispersive X-ray spectroscopy (EDX) was performed with an Oxford EDX system in combination with a Sigma VP Field Emission Scanning Electron Microscope (Carl-Zeiss AG, Germany).

High resolution transmission electron microscopy (TEM) measurements were conducted with a FEI Tecnai G^2^ 20 Transmission Electron Microscope. 15 μL of the sample solution was blotted onto lacey carbon grids (Plano). Images were acquired at an acceleration voltage of 200 kV.

BET surface areas were determined with a Quantachrome ASiQwin. Mackinawite samples were pre-treated by outgassing under high vacuum at 0 °C for six hours. Sorption isotherms were then acquired at −195.5 °C using N_2_ as adsorbate. Mössbauer spectra were obtained on a homemade spectrometer based on a RCPTM MS-96 Mössbauer spectrometer equipped with a Ritverc Co57 in a Rh-matrix source, a YAP:Ce scintillating crystal detector, and a Janis SVT-400 helium-bath cryostat. Spectra were calibrated against α-iron at room temperature and fitted using the MossWinn 4.01 program.

### Dissolution procedure for ICP-AES

The dissolution of mackinawite samples was carried out in a 100 mL Teflon lined steel reactor. The sample (around 100 to 300 mg) was placed on the bottom of the inlet and cautiously covered by water. The water was frozen in a freezer and 10 mL of aqua regia was poured on top of the ice without any contact to the mackinawite sample. The reactor was sealed and heated in an oven over night (*t* > 12 h) at 130 °C. To evaluate the efficiency of the sulfide oxidation the non-oxidized sulfur was determined in dependence of the reaction time.

## Results

### Formation mechanism

The mechanism of the reaction between iron and sulfur has already been studied by corrosion scientists.^24–32^ The reaction proceeds according to:

1 Fe^0^_(s)_ + S^0^_(s)_ → FeS_(s)_

The mechanism has been clarified to a certain degree with the most extensive report published by Schmitt in 1991.^30^ It was further extended by the work of Dowling in the following year.^27^ The combined mechanisms propose the following steps:

1. Elemental sulfur in contact with an iron surface is activated and disproportionates in water into sulfide and sulfate ions. Sulfur alone without the presence of an iron surface disproportionates considerably only in hot alkaline solutions.

2 S_8(s)_ + 8H_2_O ⇌ 6HS^−^_(aq)_ + 2SO^2−^_4(aq)_ + 10H^+^_(aq)_

2. The protons released by this reaction attack the iron surface that is covered by an oxide layer and promote the dissolution of the metal and the formation of hydrogen gas.

3 Fe_(s)_ + 2H^+^_(aq)_ ⇌ Fe^2+^_(aq)_ + H_2(g)_

3. The iron and hydrosulfide ions precipitate on the iron surface forming an initial layer of iron sulfide.

4 Fe^2+^_(aq)_ + HS^−^_(aq)_ ⇌ FeS_(s)_ + H^+^_(aq)_

4. The iron sulfide layer prevents the re-passivation of the iron surface and leads to massively enhanced corrosion termed “sulfur assisted corrosion”. As the iron sulfide layers form in small areas, pitting corrosion is predominantly observed during this stage. The released iron ions lower the local pH value and enhance the metal dissolution rate even more.

5 Fe^2+^_(aq)_ + 6H_2_O ⇌ [Fe(H_2_O)_5_(OH)]^+^_(aq)_ + H^+^_(aq)_

5. For the final step experimental evidence is missing. Dowling suggests that an electrically conducting layer of iron sulfide is formed between the iron and the sulfur surface. The dissolution of iron releases Fe^2+^ and electrons that move through the iron sulfide and react with sulfur molecules on the surface. The sulfur molecules on the surface are reduced and form polysulfide ions. When the released iron ions reach the reduced sulfur species on the sulfur surface, iron sulfide is formed extending the conducting iron sulfide layer.

6 S_*n*(s)_ + 2e− ⇌ S^2−^_*n*(aq)_

7 Fe^2+^_(aq)_ + S^2−^_*n*(aq)_ ⇌ FeS_(s)_ + S_(*n*−1)(s)_

In order to examine the final step in more detail, the reaction was monitored with a video camera and by SEM imaging in two independent experiments. The video can be found in the ESI.† For the first experiment, an iron plate was placed on top of a sand bath in a round bottom flask. Some sulfur grains were placed on the iron plate and the flask was purged with nitrogen gas for three times. Then, the flask was filled with a 0.001 M deoxygenated sodium chloride solution to half of the height of the sulfur grains. The reaction was carried out under a permanent nitrogen gas flow to exclude any oxygen contamination. The flask was kept at room temperature for 9 hours.

The video shows the formation of black iron sulfide starting at the interface between the iron plate and the sulfur grains. With time, the iron sulfide spreads over the surface of the sulfur particles. No iron sulfide is formed on the iron plates and new iron sulfide only forms as an extension of the previously formed one. This observation corresponds to the electron transport from the metal surface through the already formed iron sulfide to the reaction front to reach the elemental sulfur. The iron sulfide formation stopped at the line, where the water covers the sulfur grains. After removing the sulfur grains, the iron plate shows significant signs of pitting corrosion as can be seen from the SEM image in Fig. S2.†

In the second experiment, an iron plate was placed on a sticky SEM sample holder and molten sulfur was poured around it. The sample holder was placed in a deoxygenated sodium chloride solution on a sand bath for one day with the plate and the sulfur completely covered. After one day, the sample holder was removed and left to dry open to the atmosphere. A part of the sulfur was removed to investigate the interface.

**Fig. 1:**
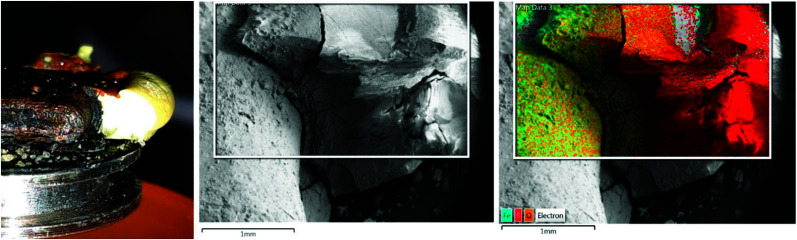
(left) SEM sample holder with an iron plate and elemental sulfur after the reaction in deoxygenated water for one day at room temperature. (middle) SEM image of the interface between iron and sulfur. (right) EDX mapping of the same area.

Images of the interface were taken from different angles and EDX single point spectra and EDX mappings were recorded. Fig. 1 shows the side view of the interface and the corresponding EDX mapping. The sulfur in contact with the iron plate is cracked but shows a smooth surface at some distance from the interface. The EDX mapping shows that the cracked area has a higher iron content, whereas in the smooth part there is only sulfur. This behavior corresponds to the formation of iron sulfide in the interface which is wettable by water. During the drying of the sample the water evaporates, and the iron sulfide contracted and cracked. As pure sulfur is not wettable by water, it stays intact and smooth.

The examination of the video and the SEM images confirm the mechanism proposed by Dowling. The electrons move through the conducting iron sulfide towards the sulfur surface. The reduction of the sulfur surface establishes an electrochemical potential that attracts the iron ions what makes them follow the electrons through the solution along the wet iron sulfide surface and form new iron sulfide at the reaction front.

### Kinetics

**Fig. 2:**
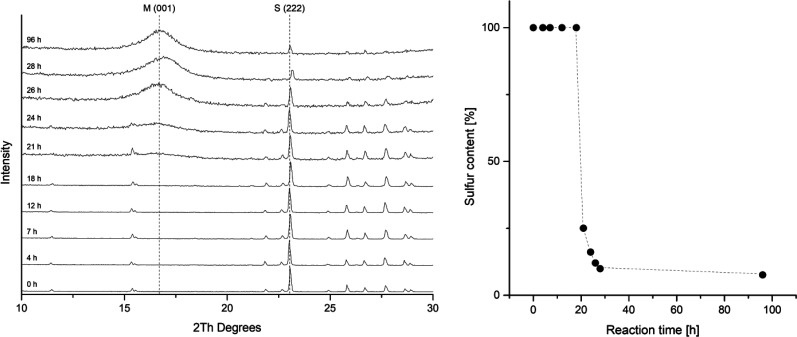
(left) Series of PXRD patterns of the reaction between iron and sulfur quenched and analyzed after different reaction times. (right) Plot of the relative sulfur content in a reaction mixture of elemental iron and sulfur in deionized water at room temperature. The sulfur content was determined by fitting the PXRD patterns using the crystal structures of elemental sulfur and mackinawite. M = mackinawite, S = sulfur. All of the other diffraction peaks can be assigned to elemental sulfur.

The rate of the corrosion of elemental iron in contact to elemental sulfur has been studied by several groups.^24,28,30,50,51^ All of these investigations report an induction period. During this period, only slow corrosion takes place, and it is assumed, that this is the time needed for the disproportionation of sulfur and the formation of the initial iron sulfide layer. After this is established, sulfur assisted corrosion takes place that is much faster. This behavior was also observed in a kinetic study tracking the reaction by PXRD (Fig. 2). The reactions in this series were carried out with iron and sulfur without additional salts. The PXRD patterns from the starting mixture shows only diffraction peaks corresponding to elemental sulfur. After 21 hours the signal to noise ratio gets worse and after 24 hours a broad hump around 5 Å *d*-spacing appears. This diffraction peak corresponds to the 001 diffraction peak of mackinawite. With further reaction time it increases but remains very broad. The sulfur diffraction peaks decrease in intensity until most of them completely disappear. A small amount of residual sulfur remains even after 96 hours, what is due to separation from the elemental iron. Little variations in the peak positions occur due to deviations of the sample height.

The PXRD patterns were fitted using the mackinawite and the sulfur crystal structure and the mass fractions were determined. In Fig. 2 the mass fractions of sulfur are plotted against the reaction time. The induction period in this setting at room temperature took around 20 hours. The following formation of mackinawite is relatively rapid and finished about 8 hours later. To increase the overall reaction rate, it is reasonable to use an additional electrolyte for a better conductivity of the solution. The addition of sodium chloride accelerates the reaction significantly. Usually, a 0.01 M solution was used to carry out the reaction with a yield of 100% after 12 hours. The pH value of the solution also showed a significant influence on the reaction rate. The higher the pH value the slower is the overall reaction and at pH values above 10.5 no reaction occurred at room temperature which may be attributed to the thickness of the passivating oxide layer on the iron surface which slows the formation of the first iron sulfide and therefore the onset of sulfur assisted corrosion.^26^

The phases that occur in the PXRD pattern are mackinawite, residual iron and sulfur without any other iron sulfide phase. A complete conversion can be achieved by thorough mixing of iron and sulfur and keeping the reactions undisturbed to prevent a separation of sulfur and iron.

### Structure and morphology

**Fig. 3:**
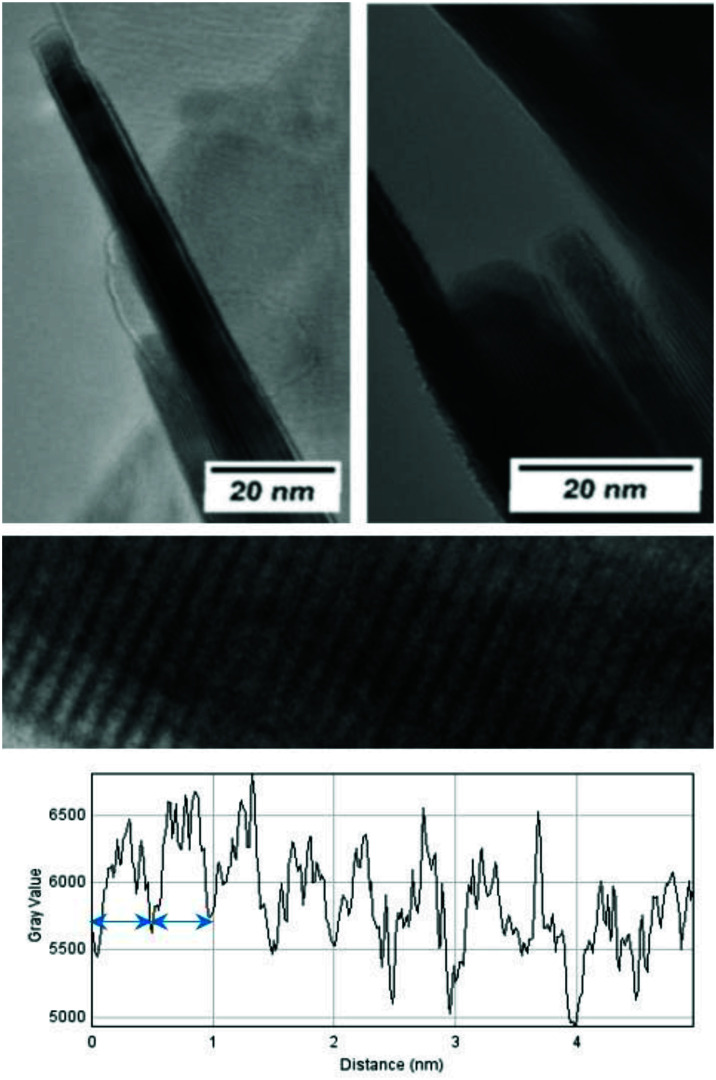
TEM investigations of mackinawite. (top) The side view of the particles clearly shows the layered structure and the small particle dimensions along the stacking direction. (bottom) The investigation of the grey value along the stacking direction indicates a d-spacing of approximately 5 Å (blue arrow).

**Fig. 4:**
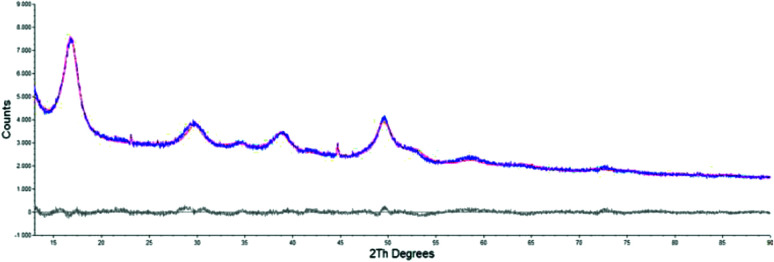
PXRD pattern of mackinawite synthesized from iron and sulfur at room temperature (blue curve). Rietveld fit using the crystal structures of mackinawite, sulfur and iron (red curve). Differential curve between experiment and fit (grey curve).

**Fig. 5:**
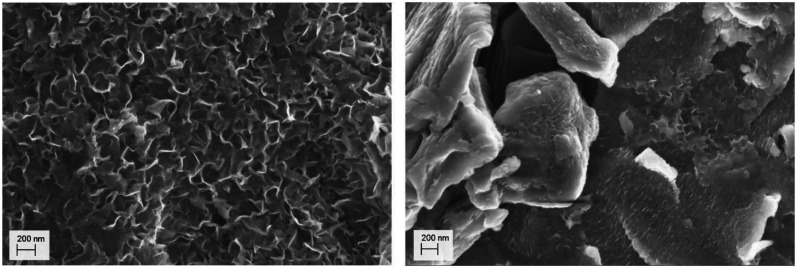
SEM images of (left) a deactivated mackinawite sample prepared from iron and sulfur in a 0.01 M sodium chloride solution for 12 h and (right) stored open to the atmosphere for one month at room temperature.

The morphological and structural properties of the mackinawite produced from the elements were determined by PXRD and TEM/SEM imaging. Representative PXRD patterns are shown in Fig. 4 and S5.† A Rietveld refinement was carried out as described in the methods section. The fitting parameters are summarized in Table TS1 in the ESI.† The Rietveld-refinement with *R*_wp_ = 0.02815 using the mackinawite crystal structure gives lattice parameters of *a* = 3.6574 ± 0.0007 Å and *c* = 5.2717 ± 0.011 Å. SEM and TEM images show curved platelets that form micrometer sized aggregates (Fig. 3 and 5). The particles have a diameter of hundreds of nanometers with a thickness of only 5 to 30 nm. The fitted size parameter is reasonable as it corresponds to a particle size less of than 30 nm. The spacings of the lattice fringes in TEM images could be determined to be around 0.5 nm, corresponding to the interlayer spacing of the mackinawite crystal structure.^52^ The c-parameter with *c* = 5.27 Å is slightly bigger compared to the one of highly ordered mackinawite with *c* = 5.03 Å.^47^ The BET surface area was determined to be between 40 m^2^ g^−1^ and 80 m^2^ g^−1^ using nitrogen gas.

The mackinawite particles formed in the reaction of wet elemental iron with elemental sulfur at room temperature are very similar to mackinawite particles obtained by precipitation.^53^ Therefore, this reaction may be considered as a precipitation at very constant concentrations reproducibly leading to the formation of nanoparticulate mackinawite.

## Composition

The compositions of the mackinawite samples synthesized from the elements were determined with ICP-AES. The main difficulty in the acidic dissolution of mackinawite is the formation of H_2_S. Rickard *et al.* used a sophisticated dissolution apparatus in which the H_2_S was trapped by Cu^2+^ and analyzed independently of Fe^2+^.^54^ The oxidation of the sulfide ions is a different suitable approach that was followed in this work. The mackinawite samples were placed in a 100 mL Teflon lined steel reactor and covered with water. The water was carefully frozen and aqua regia was poured on top of the ice without contact to the iron sulfide. The reactor was sealed and heated in an oven overnight at 130 °C until the sample was completely dissolved and all sulfide was oxidized to sulfate. The sulfide oxidation is a rather slow process and was found to be completed after 12 hours at 130 °C (see Fig. S4†). Including the experimental error of the method itself, the iron–sulfur-ratio was determined to be Fe : S = (1.010 ± 0.004) : (1.000 ± 0.003) with no residual elemental iron or sulfur or any other phase visible in the PXRD pattern. In the applied method of synthesis, minor amounts of elemental iron or sulfur may be hidden in the background of the PXRD pattern and could influence the composition that is determined. In this way, the accuracy of the composition is limited more by the PXRD measurements than by the ICP-AES method and the composition could have greater errors than given. The existence of residual elemental iron was checked with a strong magnet and samples were only analyzed when no more magnetic material was found in the reaction mixture. The water content of the samples was calculated by difference neglecting the sodium content as not significant. The samples that were dried by purging with cold nitrogen gas showed high water contents leading to compositions from FeS·0.85H_2_O to FeS·1.46H_2_O. Heating the samples to 80 °C under a nitrogen gas atmosphere leads to the release of the water but not to a change in the structure.

### Pyrophoricity

Nanoparticulate mackinawite is quite stable when wet but highly pyrophoric in the dry state. The oxidation reaction is very exothermic and leads to the formation of lepidocrocite and SO_2_ as determined by PXRD and GC-MS analysis.

8 2FeS_(s)_ + 3.5O_2(g)_ + H_2_O → 2FeO(OH)_(s)_ + 2SO_2(g)_

To analyze dried samples, it was necessary to cautiously oxidize the particle surfaces and prevent their spontaneous ignition. This was done by short contact times to air and cooling by flowing nitrogen gas again. After 3 to 6 cycles the mackinawite samples were stable and could be handled open to the atmosphere without ignition. The oxidation of the surface of the particles was neither visible by PXRD nor by SEM imaging, but it was observed that the particles stored open to the ambient atmosphere for several days get covered with a smooth layer that reduces the available surface area determined by BET from around 80 m^2^ g^−1^ to only 3 m^2^ g^−1^ (Fig. 5). This layer consists of greigite as determined by PXRD analysis.

### Mössbauer spectroscopy

**Fig. 6:**
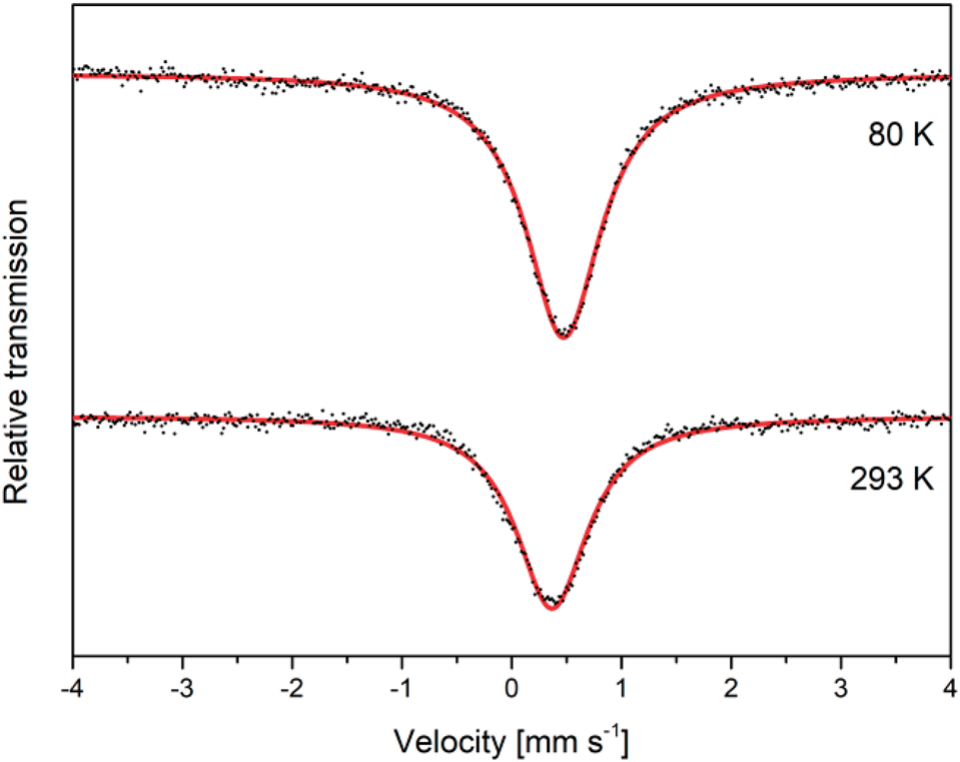
Mössbauer spectra for a deactivated mackinawite sample, produced by the elemental route, measured at different temperatures.

Mössbauer analysis was carried out on a mackinawite sample prepared from elemental iron and sulfur in a 10^−3^ M NaCl solution at room temperature overnight. The sample was cautiously oxidized and the spectra were recorded at room temperature and 80 K over a velocity range of (4 mm s^−1^) with reference to metallic iron. The observed Mössbauer spectra could both be well fitted as singlets, which correspond to the low spin Fe^2+^ ions (Fig. 6). The observed isomer shifts of 0.37 mm s^−1^ and 0.47 mm s^−1^, respectively, are in close agreement with previously reported values for synthetic mackinawite (Table 1).

**Table 1:** Center shifts (*δ*) for synthetic mackinawite at different temperatures (*T*), relative to iron, from the literature and this work

Reference	*T* (K)	*δ* (mm s^−1^)	Rel. area (%)
Schröder, 2020 (ref. 55)	293	0.37	100
	4	0.49	
Boursiqout, 2001 (ref. 56)	295	0.42	52
Vaughan, 1971 (ref. 57)	4	0.2	100
This work	292	0.37	100
	80	0.47	

When mackinawite is oxidized by air, the occurrence of additional doublets has been reported. They are corresponding either to Fe^3+^ ions in tetrahedral sites or Fe^2+^ in close proximity to Fe^3+^.^56^ The analyzed sample did not show any additional signals even at 80 K and we therefore assume that the particles are nearly free from Fe^3+^ ions, even after the oxidation of the surface. Probably only the most reactive surface sites are oxidized due to the contact with air at room temperature and the core of the particles remains nearly unchanged.

### Model of charged layers

We propose a new model for the structure and composition of nano-mackinawite that is able to explain some inconsistent observations reported in the literature. The reaction of elemental iron with elemental sulfur proceeds in distilled water in an inert atmosphere at room temperature and therefore offers completely new opportunities to study the formation of nano-mackinawite as the influence of the surrounding solution can be addressed independently.

We call the elaborated model “charged layers” model as it divides mackinawite particles into two groups: mackinawite with negatively charged layers on one hand and mackinawite without significant negative charge in its interior on the other. The non-charged mackinawite is characterized by a relatively sharp 001 diffraction peak in X-ray diffraction and has an interlayer spacing close to 5.03 Å (*e.g.* Lennie, 1995). The charged mackinawite is characterized by a very broad 001 diffraction peak due to pronounced strain effects and an interlayer spacing greater than 5.05 Å up to values of 5.7 Å (Fig. S5†).

Mackinawite with the characteristics of both states has been described in previous works on the precipitation of Fe^2+^ ions and sulfide ions as can be seen in Table 2. It shows the compositions of precipitated mackinawite and the determined *d*-values of the 001 diffraction peaks given in some reports on mackinawite.

**Table 2:** Mackinawite compositions (Fe : S) and *d* (001) values found in the literature. n.d. = not determined

Reference	Experiment	Fe : S	*d* [Å]
Berner, 1964 (ref. 63)	Fe + H_2_S (RT)	1.05 : 1	5.03
Rickard, 1969 (ref. 54)	Fe^2+^ + HS^−^ (RT)	1 : 1,1	5.03
Lennie, 1995 (ref. 47)	Fe + HAc + Na_2_S (RT)	1.008 : 1	5.03
Mullet, 2002 (ref. 64)	Fe + HAc + Na_2_S (RT)	1 : 1	5.05
Wolthers, 2003 (ref. 61)	Fe^2+^ + Na_2_S (RT)	n.d.	5.48
Michel, 2005 (ref. 65)	Fe^2+^ + Na_2_S (RT)	n.d.	5.0X
Rickard, 2006 (ref. 46)	Fe^2+^ + Na_2_S (RT)	1 : 1	n.d.
Ohfuji, 2006 (ref. 53)	Fe^2+^ + Na_2_S (RT)	n.d.	5.19 (wet)
			5.08 (dried)
Jeong, 2008 (ref. 66)	Fe^2+^ + Na_2_S (RT)	n.d.	5.20
Bourdoiseau, 2008 (ref. 67)	Fe^2+^ + Na_2_S (RT)	n.d.	5.7
Bourdoiseau, 2011 (ref. 10)	Fe^2+^ + Na_2_S (RT)	n.d.	5.05
Csákberényi-Malasics, 2012 (ref. 7)	Fe^2+^ + C_2_H_5_NS (RT)	n.d.	5.88
	Fe^2+^ + C_2_H_5_NS (120 °C)	n.d.	5.03
This work	Fe + S (RT)	1 : 1	5.26–5.29
	Fe + S (80 °C)		5.07–5.29

We could not identify the conditions that preferably lead to the formation of either state. There are many parameters to be considered like local concentrations of S^2−^, Fe^2+^, pH value, concentrations of counter ions like Na^+^, NH_4_^+^, NO_3_^−^, SO_4_^2−^, Cl^−^, temperature, purity of starting materials, oxygen contamination, mixing rate and any aging procedures.

The charged mackinawite is considered to consist of nano sheets with a mackinawite structure with iron vacancies. Thus, the layers are negatively charged and repel each other leading to an increased interlayer spacing. Mackinawite-like layers with iron vacancies that lead to a negative charge have been described in publications regarding the mineral tochilinite and synthetic analogues.^21,58,59^ As the obtained mackinawite is stoichiometric in regard of iron and sulfur it is assumed that the released iron ions are adsorbed onto the surface of the particles and balance the anionic charge. The vacancies may not be distributed evenly in the sheets what leads to curvature of the particles as can be seen in SEM images and peak broadening in PXRD.

### Interlayer spacing

Increased interlayer spacings in mackinawite have been reported to be caused by interstitial water or intercalated ions, but both explanations do not fit to our observations.^60,61^ In our experiments, the interlayer spacing did not change by drying, neither at room temperature in a nitrogen gas flow after successive washing with ethanol, acetone, diethyl ether and absolute THF, nor at 80 °C at reduced pressure.

The intercalation of Fe^2+^ or Na^+^ between the anionic mackinawite sheets would be comparable to the behavior of clays with anionic layers. The interlayer spacing of these clays strongly depends on the radius of the intercalated ions and is subject to swelling.^62^ However, the interlayer spacing of nano-mackinawite was constant independent of the sodium and water content of the samples. If intercalated Fe^2+^ would be replaced by Na^+^, a change in the interlayer spacing would be expected as the ionic radius of Na^+^ is much larger.

### Adsorbed cations on the mackinawite surface

It was experimentally shown that the addition of sodium chloride to a mackinawite suspension leads to the release of Fe^2+^ into the solution. The released iron ions can be precipitated by additional sulfide ions to form iron sulfide again. The addition of (NH_4_)_2_Fe(SO_4_)_2_ did not lead to any precipitate. This behavior of mackinawite has not been described before and cannot be explained based on previous reports. It has been reported that transition metals whose sulfides are less soluble than mackinawite can enter the mackinawite lattice and substitute Fe^2+^, leading to their release into the soultion.^68–70^ Because sodium ions should not be able to substitute iron ions from the mackinawite lattice, the replaced iron ions are expected to be adsorbed onto the particle surfaces and mainly attracted by weak electrostatic forces.

**Fig. 7:**
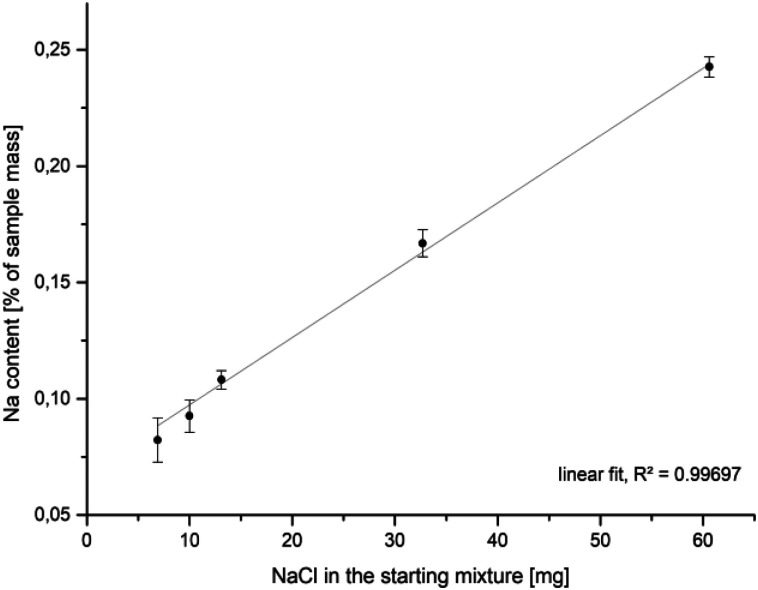
Na-content in mackinawite samples (ICP-AES) synthesized from iron, sulfur and sodium chloride in water at room temperature *versus* the amount of sodium chloride in the starting mixture.

Compositional analyses of mackinawite samples synthesized with increasing amounts of sodium chloride show an increasing amount of sodium in the final washed and dried sample. The data can be fitted by linear regression what is reasonable if the surface Fe^2+^–Na^+^-exchange depends solely on the sodium chloride concentration at a constant temperature (Fig. 7). The released amount of iron ions is too little to be confidently determined by the applied ICP-AES method, but the pH of the solution gives a simple way to access them indirectly. The addition of increasing amounts of sodium chloride to mackinawite synthesized from the elements leads to a lower pH at the end of the reaction which is a consequence of a rising concentration of Fe^2+^ ions that act as a weak acid and release protons upon hydration (Table 3).

**Table 3:** pH values of the reaction solution after mackinawite synthesis from iron, sulfur and sodium chloride at different sodium chloride concentrations

*c*(NaCl) [mol L^−1^]	pH
0.0127	5.67
0.1496	4.48
0.2750	3.68

It has been shown experimentally that a higher sodium chloride concentration leads to a lower pH value of the solution. This can be explained by an equilibrium process. The sodium ions reversibly replace the surface iron ions whereby the ratio of replaced to surface adsorbed Fe^2+^ depends on the sodium concentration.

9 Fe_(s)_ + S_(s)_ → [Fe_(1−*x*)_S]^2*x*−^ + *x*Fe^2+^_(ads)_

10 Fe^2+^_(ads)_ + 2Na^+^_(aq)_ ⇌ 2Na^+^_(ads)_ + Fe^2+^_(aq)_

11 Fe^2+^_(aq)_ + 6H_2_O ⇌ [Fe(H_2_O)_5_(OH)]^+^_(aq)_ + H^+^_(aq)_

### Aging and particle growth

It is expected that particle growth with time will inevitably lead to less charged mackinawite because the difference of charge on the surface of the particles compared to the interior creates an electrochemical potential. This potential rises upon crystallite growth as with every new layer more vacancies and corresponding cations are added to the same particle and more charge is accumulated. The rising potential forces the following layers to have ever fewer vacancies which is why the charged mackinawite is only expected to exist with very small particle sizes. It is reasonable to expect that the particles can become uncharged by oxidation, but this has not been achieved in our experiments. Nevertheless, previous reports support that the precipitation of mackinawite leads to the formation of the non-charged form, if any oxidants are present. Most syntheses reported have been carried out under strictly inert conditions without any oxidants present but the actual conditions may have been misjudged. For example, Boursiquot *et al.* state that their “Mackinawite synthesis was carried out under carefully controlled oxygen-free conditions.”^56^ Still, their Mössbauer data clearly show that the obtained mackinawite is oxidized and contains a significant amount of Fe^3+^. This can be explained by the presence of sulfate or acetate ions which may act as oxidants during the mackinawite synthesis. The absence of oxygen may not be sufficient for the synthesis of Fe^3+^-free mackinawite. It is reasonable to assume, that the absence of any oxidants may be a prerequisite for the formation of charged mackinawite with an interlayer spacing greater than 5.05 Å.

**Fig. 8:**
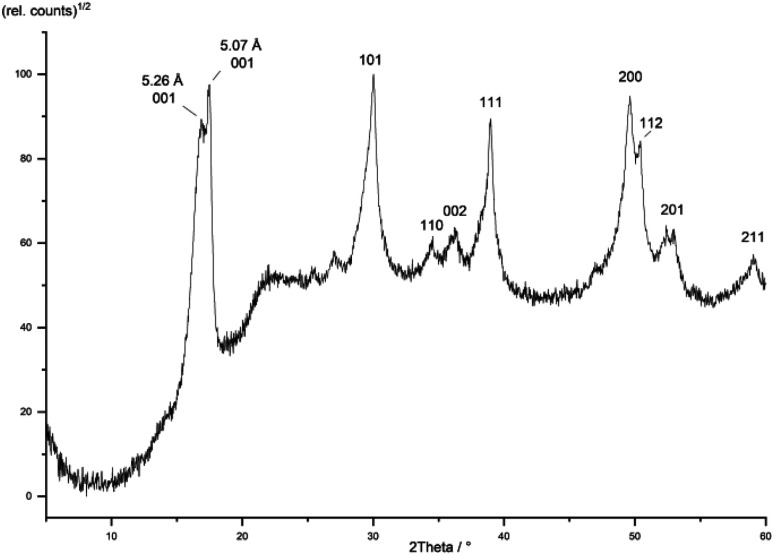
PXRD pattern of a mackinawite sample aged at 80 °C for 7 days *hkl*-assignment according to ref. 49.

The model assumes, that the charged mackinawite will transform into the noncharged form upon aging. A sample of mackinawite aged in a solution with a low sodium chloride concentration (10^−3^ mol L^−1^) at 80 °C for seven days shows the appearance of a 001 diffraction peak at *d* = 5.07 Å and a change in the shape of the other diffraction peaks that is caused by the overlap of broad and narrow peaks at nearly the same *d*-values (Fig. 8). This “tailing” of the diffraction peaks is considered to be a direct consequence of the change of the interlayer space and the transformation of charged into noncharged mackinawite upon aging.

The transformation of aged mackinawite has also been reported multiple times in the literature. The aging of freshly precipitated mackinawite leads simultaneously to lower interlayer distances and smaller widths at half maximum of the 001 diffraction peak.^7,71^ There are no reports on pure mackinawite samples with large particles and an increased interlayer spacing. In the frequently quoted work of Wolthers *et al.* from 2003 (ref. 61) an investigation of nanoparticulate mackinawite with low angle X-ray diffraction (LAXRD) was carried out. The LAXRD pattern showed very broad diffraction peaks and a *d*-value for the 001 diffraction peak greater than 5.5 Å. It was fitted using two mackinawite structures with different interlayer spacings as the fit was very poor when using only one set of lattice parameters. The authors stated that: “The fact that the patterns could be fitted with a minimum of two peak sets indicates that the material is a mixture of at least two disordered mackinawite phases, referred to as *M*_kA_ and *M*_kB_, with varying *d*-spacing and crystallinity”.^61^ It is apparent, that their sample may have contained multiple or even a nearly infinite number of different mackinawite particles with different interlayer spacings. There is no reason to assume, that there are only two distinct forms *M*_kA_ and *M*_kB_. This observation may be explained by the charged layers model, as it is expected that there is a distribution of different interlayer spacings and as there is a minimum value of around 5 Å, an asymmetric distribution of interlayer spacings leads to the observed “tailing”.

### Point of zero charge

More support for this model can be found in the adsorption behavior of nano mackinawite and the determined points of zero charge. The adsorption of Cd^2+^ ions on the mackinawite surface follows an ion exchange mechanism replacing iron ions from the mackinawite surface as shown by Mustafa *et al.* 2010.^13^ Such an adsorption behavior would be expected for charged mackinawite particles. The point of zero charge of mackinawite was determined to be around pH_pzc_ = 3 on the one hand and around pH_pzc_ ≈ 7.5–8 on the other.^72–74^ This discrepancy may also arise from the different behaviors of charged and noncharged mackinawite. The mackinawite particles in these publications were not analyzed sufficiently to establish a correlation between the pH_pzc_ and the structure. For charged mackinawite it is to be expected nonetheless, that the positively charged metal ions on the surface lead to a pH_pzc_ above 7 or at least higher than the noncharged one. The noncharged mackinawite surface is dominated by sulfide groups and should therefore show a pH_pzc_ around 3 like pyrite and pyrrhotite.

## Conclusions

The synthesis of mackinawite from the elements at low temperatures is a convenient way to obtain mackinawite nanoparticles that resemble the characteristics of mackinawite synthesized by precipitation. The advantages of the mackinawite synthesis from the elements include:

• No use of sensitive chemicals like Fe^2+^ salts or sulfides that are more difficult to purchase and store without any traces of oxidation.

• No use of toxic and environmentally harmful sulfur sources like Na_2_S or H_2_S.

• Much better control over the reaction conditions as any additional salt can be introduced very precisely prior to the reaction making this synthesis more reliable and reproducible than conventional precipitation reactions.

Furthermore, a convenient method was developed to dissolve mackinawite samples for elemental analysis by ICP-AES. A new model for the structure of nanoparticulate mackinawite was developed that proposes negatively charged particles that contain a certain amount of iron vacancies. This charge is compensated for by Fe^2+^ adsorbed onto the surface which can be exchanged by other cations like Na^+^. The negative charge of the mackinawite layers leads to their repulsion and an increased interlayer spacing. Upon aging and particle growth, the interlayer spacing decreases and the particles show a higher degree of crystallinity.

Mackinawite has been shown to be of use for many different applications like wastewater treatment or as catalyst. However, due to its inevitable reaction with O_2_ the implementation of possible applications is limited. Our investigations show, that mackinawite can be stabilized without changing the basic structure of the particles by only oxidizing the surface. This passivating layer can recrystallize under appropriate conditions, revealing the reactive mackinawite surface again. This finding represents an convenient way to store mackinawite and handle its high reducing power.

Furthermore, the reaction conditions of the elemental route reassemble those likely present on the early earth's surface. In the literature concerned with prebiotic chemistry, mackinawite was only considered to be present in significant amounts at hydrothermal sites, where it precipitated from HS^−^ and Fe^2+^. The formation from the elements probably led to mackinawite formation on the surface of the early earth. Compared to submarine systems, the surface supports a wider variety of reaction conditions like wet–dry cycles and photochemistry as well as a much easier access to atmospheric gases and material delivered by cosmic impacts. As many of these scenarios are still to be investigated, the principal idea of mackinawite being present on the surface of the early earth could give rise for a novel approach on the early chemical evolution shortly after earth's formation.

## Conflicts of interest

There are no conflicts to declare.

## Supplementary Material

RA-011-D1RA03705F-s001
