# Supplementary material for: Mackinawite formation from elemental iron and sulfur
Source: RSC Adv. 2021 Oct 1;11(51):32464–75. doi: 10.1039/d1ra03705f (PMC9041996; doi:10.1039/d1ra03705f)
Supplement: RA-011-D1RA03705F-s001 [file RA-011-D1RA03705F-s001.pdf]

Electronic Supplementary Material (ESI) for RSC Advances.
This journal is © The Royal Society of Chemistry 2021

Supporting Information for

Robert Bolney, Mario Grosch, Mario Winkler, Joris van Slageren, Wolfgang Weigand and Christian Robl

Mackinawite formation from elemental iron and sulfur

Figure S1: SEM image of the used iron powder. EDX analyses shows the presence of iron and very small fractions of carbon, oxygen and sulfur.

Figure S2: SEM image of an iron plate after the reaction with sulfur at room temperature in deoxygenated water.

Figure S3: EDX analysis of a washed mackinawite sample free from chloride.

Figure S4: Plot of the not oxidized sulfur content of reference mackinawite samples versus the reaction time at 130°C from mackinawite dissolution experiments.

Figure S5: PXRD pattern of a typical deactivated mackinawite sample obtained from the reaction between iron and sulfur in a 0.01 M sodium chloride solution at room temperature after 12 hours reaction time without any residual iron or sulfur.

Table TS1: Fitting parameters of the Rietveld fit of a PXRD of mackinawite prepared from elemental iron and sulfur.

link to video

https://upload.uni-jena.de/data/601276cfa9cf47.37056468/64fach.mp4

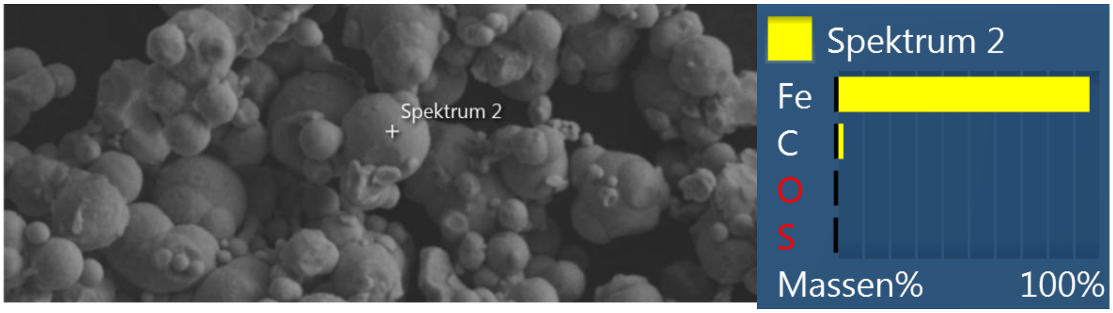

Figure S1: (left) SEM image of the used iron powder. (right) EDX analyses showing the presence of iron and very small fractions of carbon, oxygen and sulfur.

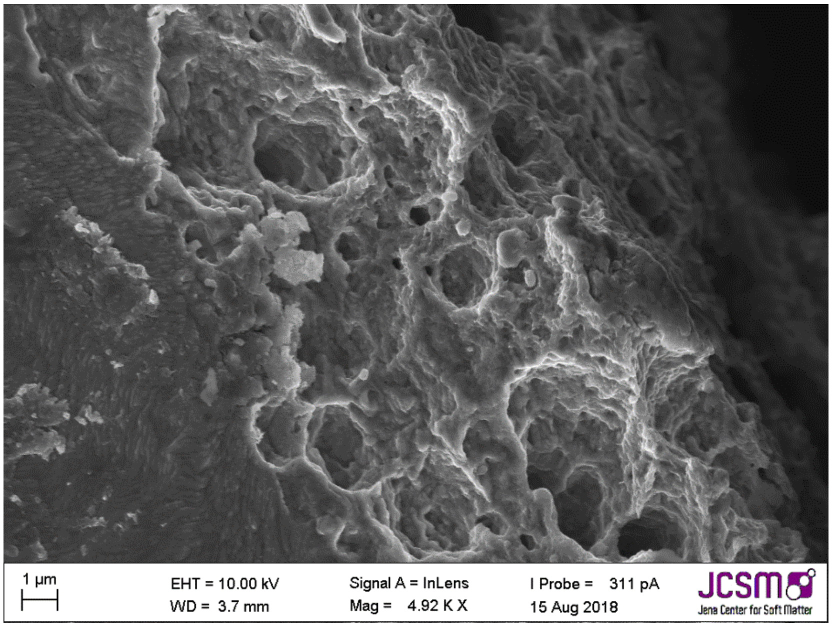

Figure S2: SEM image of an iron plate after the reaction with sulfur at room temperature in deoxygenated water.

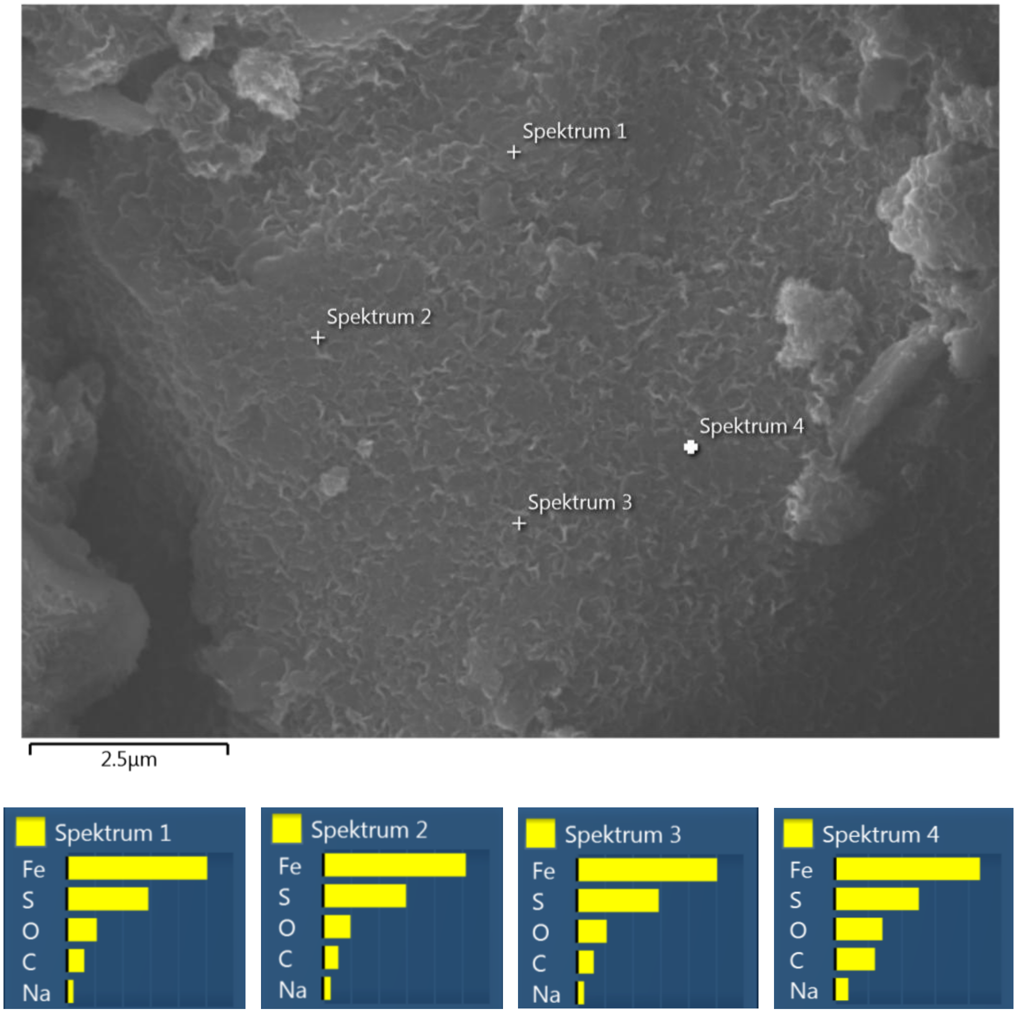

Figure S3: EDX analysis of a washed mackinawite sample free from chloride.

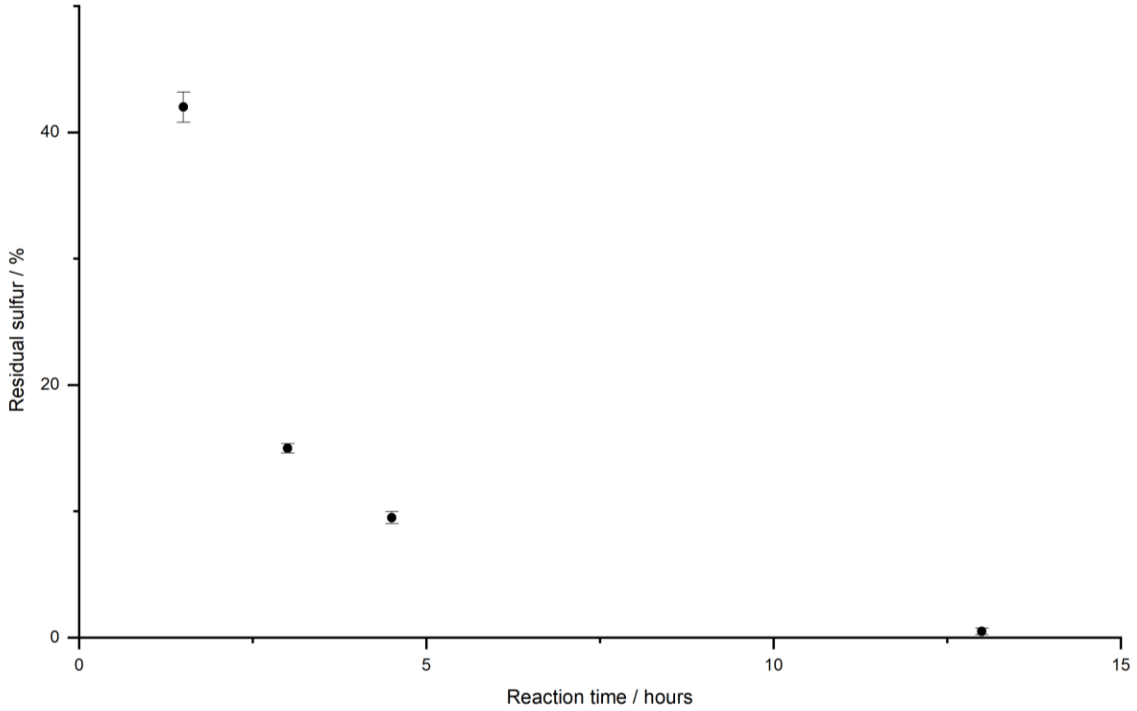

Figure S4: Plot of the not oxidized sulfur content of reference mackinawite samples versus the reaction time at 130°C from mackinawite dissolution experiments.

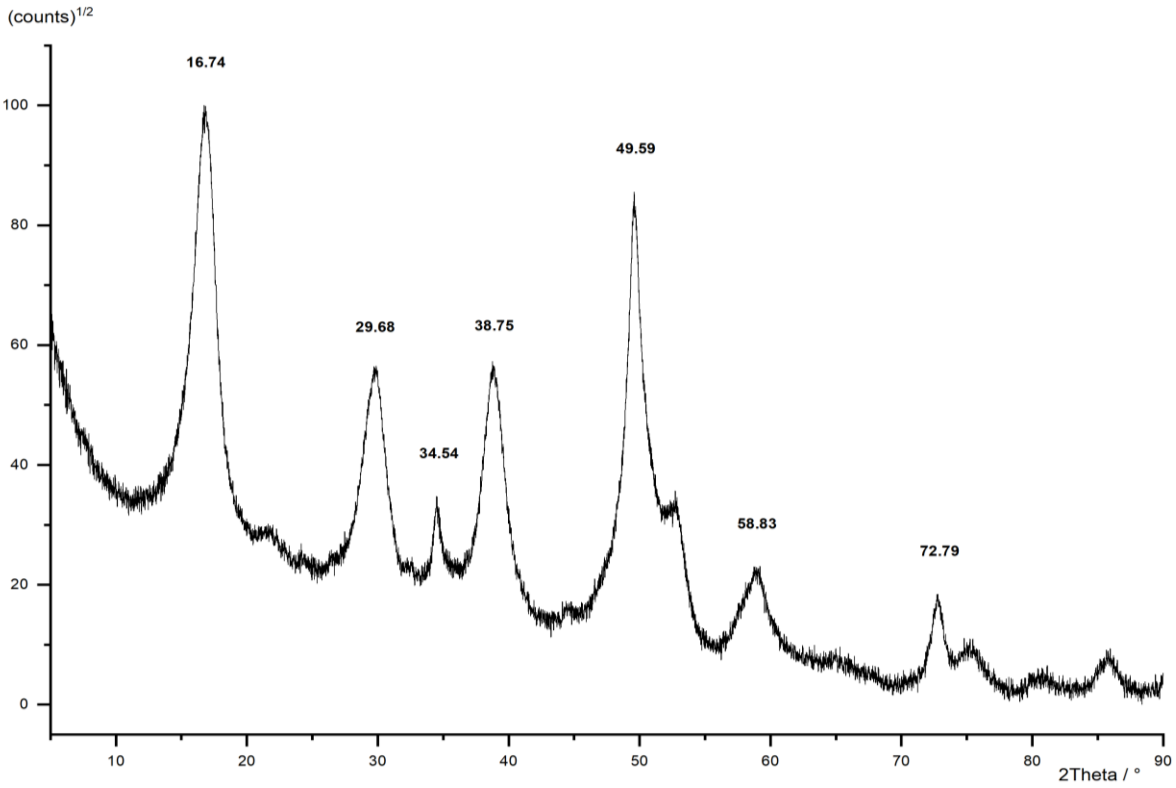

Figure S5: PXRD pattern of a typical deactivated mackinawite sample obtained from the reaction between iron and sulfur in a 0.01 M sodium chloride solution at room temperature after 12 hours reaction time without any residual iron or sulfur.

Table TS1: Fitting parameter of the Rietveld fit of a PXRD of mackinawite prepared from elemental iron and sulfur.

Parameter	Value / explanation
R_{wp}	0.02815
χ^2	1.51
Number of independent parameters	9
a	3.6574 ± 0.0007 Å
c	5.2717 ± 0.011 Å
Temperature factor Fe^{2+} and S^{2-}	0.297 ± 0.075
Size parameter	Additional gaussian convolution as a function of 1/cos(θ)
Strain parameter	Additional gaussian convolution as a function of tan(θ)
Preferred orientation along 001	Reduction of intensities along 001 due to the flat particle shape
Scale parameters (3 Phases)	Relative scale of mackinawite, iron and sulfur
